# Mining and expression analysis of candidate genes involved in regulating the chilling requirement fulfillment of *Paeonia lactiflora* ‘Hang Baishao’

**DOI:** 10.1186/s12870-017-1205-1

**Published:** 2017-12-22

**Authors:** Jiaping Zhang, Danqing Li, Xiaohua Shi, Dong Zhang, Shuai Qiu, Jianfen Wei, Jiao Zhang, Jianghua Zhou, Kaiyuan Zhu, Yiping Xia

**Affiliations:** 1 Institute of Landscape Architecture, College of Agriculture & Biotechnology, Zhejiang University, Hangzhou, 310058 China; 2 Research & Development Centre of Flower, Zhejiang Academy of Agricultural Sciences, Hangzhou, 311202 China; 3 Research & Development Center, Hangzhou Landscaping Incorporated, Hangzhou, 310020 China

**Keywords:** Chilling requirement, Bud dormancy, Gene mining, Temperature response, Photoperiod response, *SUPPRESSPOR OF OVEREXPRESSION OF CONSTANS1* (*SOC1*), Herbaceous peony (*Paeonia lactiflora*)

## Abstract

**Background:**

The artificial enlargement of the planting area and ecological amplitude of ornamentals for horticultural and landscape applications are significant. Herbaceous peony (*Paeonia lactiflora* Pall.) is a world-famous ornamental with attractive and fragrant flowers and is mainly planted in temperate and cool areas. Comparatively higher winter temperatures in the subtropical and tropical Northern Hemisphere result in a deficit of chilling accumulation for bud dormancy release, which severely hinders “The southward plantation of herbaceous peony”. Studies on the dormancy, chilling requirement (CR) and relevant molecular mechanisms of peony are needed to enhance our ability to extend the range of this valuable horticultural species.

**Results:**

Based on natural and artificial chilling experiments, and chilling hour (CH) and chilling unit (CU) evaluation systems, the lowest CR of ‘Hang Baishao’ was between 504.00 and 672.00 CHs and the optimal CR was 672.00 CHs and 856.08 CUs for achieving strong sprouting, growth and flowering performance. Transcriptome sequencing and gene identification by RNA-Seq were performed on ‘Hang Baishao’ buds during the dormancy and sprouting periods. Six gene libraries were constructed, and 66 temperature- and photoperiod-associated unigenes were identified as the potential candidate genes that may regulate or possibly determine CR characteristics. The difference in the expression patterns of *SUPPRESSPOR OF OVEREXPRESSION OF CONSTANS1* (*SOC1*) between the winters of 2012–2013 and 2015–2016, and the difference of CR fulfillment periods also between these two winters represented the interesting congruent relationships. This correlation was also observed for *WRKY DNA-BINDING PROTEIN 33* (*WRKY 33*).

**Conclusions:**

Combined with the results acquired from all of experiments, ‘Hang Baishao’ was confirmed to be a superb peony resource that have significantly low CR characteristics. The two genes of *SOC1* and *WRKY33* are likely involved in determining the CR amount and fulfillment period of ‘Hang Baishao’. *HEAT SHOCK PROTEIN*, *OSMOTIN* and *TIMING OF CAB EXPRESSION 1* also deserve attention for the CR research. This study could contribute to the knowledge of the deep factors and mechanisms that regulate CR characteristics, and may be beneficial for breeding new germplasms that have low CRs for landscape or horticulture applications in subtropical regions.

**Electronic supplementary material:**

The online version of this article (10.1186/s12870-017-1205-1) contains supplementary material, which is available to authorized users.

## Background

Herbaceous peony (*Paeonia lactiflora* Pall.) is a perennial geophyte that is indigenous to China and known for its extremely fascinating and vibrantly colorful flowers [1]. Herbaceous peony is mainly cultivated in temperate or cool areas of many countries in the Northern Hemisphere, including the UK, Netherlands, USA and Japan [2, 3]. The goal of promoting the landscape and garden uses of this species in subtropical and tropical zones is intriguing but not easy to achieve. “The southward plantation of herbaceous peony” is a classical and historic problem in the field of ornamental horticulture in China. This problem has received much hypothetical attention for several decades but still lacked practice and relevant in-depth scientific investigation.

The underground renewal buds of herbaceous peony must undergo a critical chilling period to break dormancy and re-initiate sprouting, growth and subsequent flowering [4]. Hot or muggy climatic conditions severely impede the natural distribution and cultivation of herbaceous peony in subtropical and tropical areas of the Northern Hemisphere. Nowadays, herb peony has not been planted widely or intensively in any south region of Yangtze River in China. Taking the Zhejiang Province for example, this is an economically developed province located in the southern region of the Yangtze Delta, which is located in the subtropical area of China. This region is significantly hotter than the northern region of China throughout the entire year but especially during winter due to the comparatively lower latitudes of the region (N 27°12′-31°31′). Another reason involves global warming trends during the past several decades [5]. These two reasons have led to severe chilling deficit regarding the bud dormancy release of *P. lactiflora*; this deficit further causes abnormal bud breakage and limited or nonexistent flowering, which greatly limits its sustainable production and application in Zhejiang [6–8]. Thus, the breeding of cultivars that have a low chilling requirement (CR) will sufficiently promote the landscape or garden use of *P. lactiflora* in subtropical and potentially tropical zones.

Evaluating the CR for bud dormancy release and understanding the essence in regulating or directly determining the CR characteristics of herbaceous peony are both indispensable for achieving the goal of “The southward plantation of herbaceous peony”. During 2001 to 2007, Fulton et al. (2001), Hall et al. (2001) and Kamenetsky et al. (2003a) studied the CR and forcing temperature responses of *Paeonia* spp.; the emphases of these authors differed, but their results are classical references and have inspired many subsequent *Paeonia* studies [9–11]. However, the materials used in these studies were all traditional herbaceous peony germplasms that have distinct preferences for cool climatic conditions. The systematic study of specific *Paeonia* germplasms that have high adaptability and low CR characteristics under warm winter conditions has not been reported. In addition, molecular studies, such as the discovery and expression of key genes closely related to the process of bud dormancy and CR fulfillment, also need to be performed [7].

**Fig. 1:**
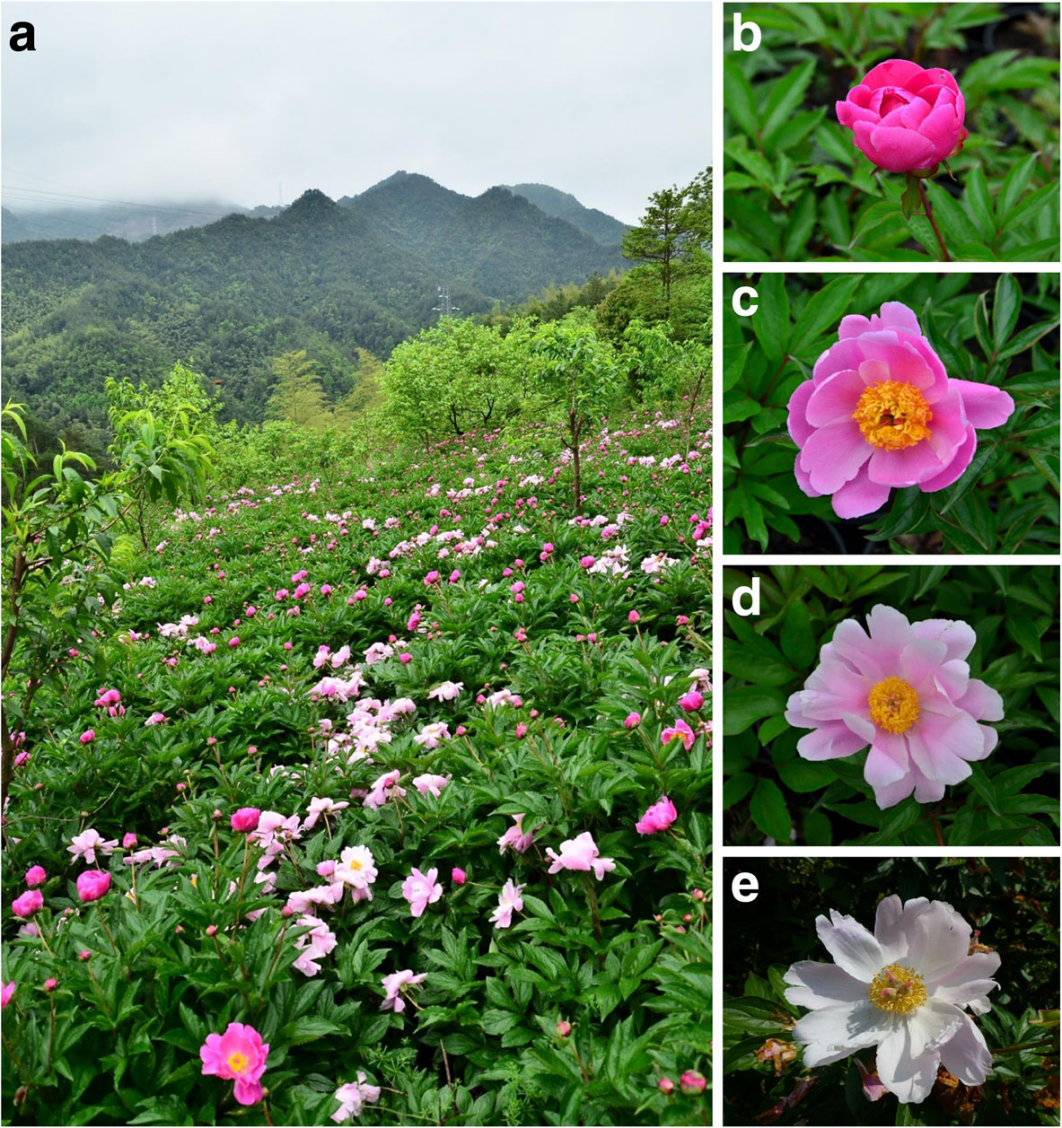
*P. lactiflora* ‘Hang Baishao’ with attractive and color-changing flowers. **a** Intensive plantation and spectacular scenery of ‘Hang Baishao’ in Jinhua City in the middle area of Zhejiang. **b** The pink purple flower in early flowering stage. **c-d** The pink or pink white flower in full flowering stage. **e** The pink white or white flower in end flowering stage

*Paeonia lactiflora* ‘Hang Baishao’ is a unique local germplasm that is cultivated in subtropical areas of China together with other species and cultivars of the Paeoniaceae family. ‘Hang Baishao’ has been planted in the middle area of Zhejiang for more than 1800 years as a traditional Chinese medicine and also as an ornamental that has attractive, color-changing flowers (Fig. 1). ‘Hang Baishao’ has better growth and flowering performance, less CR for releasing dormancy, and stronger resistance to the local hot and humid climate in Zhejiang compared with other northern *Paeonia* germplasms; therefore, ‘Hang Baishao’ should be a good parent for breeding new cultivars with high adaptability to warm winter climates. In this study, we adopted ‘Hang Baishao’ as a pioneer material to study its unique CR characteristics (amount and fulfillment period) for bud dormancy release and sprouting, performed transcriptome sequencing and quantitative RT-PCR (qRT-PCR) in plants grown under two different warm winter conditions, and identified potentially important genes that may be involved in influencing or determining CR characteristics.

Traditional studies have often focused on identifying genes that promote or inhibit the phases of paradormancy, endodormancy or ecodormancy, and analyzing the related metabolism processes or signal transduction pathways, such as chromatin remodeling and carbohydrate or phytohormone metabolism [7, 12–20]. However, in this study, we attached more importance to another perspective: the mining of genes that could regulate or directly determine the inherent CR amount/fulfillment period of herbaceous peony. This study expanded our understanding of the bud dormancy process, CR characteristics, and the potential function of CR-associated genes in a new perspective. Furthermore, this study could also provide a theoretical basis for breeding new cultivars of herbaceous peony that have strong adaptability to warm winter in order to achieve the ultimate goal of “The southward plantation of herbaceous peony”.

## Methods

### Plant material

Divided crowns of five-year-old *P. lactiflora* ‘Hang Baishao’ plants were introduced from Pan’an County (E 120°17′-120°47′, N 28°49′-29°19′) of Jinhua City in Zhejiang Province (Fig. 1) and planted in the Perennial Flower Resources Garden of Zhejiang University in Hangzhou (E 118°21′-120°30′, N 29°11′-30°33′). All the crowns were planted in one-gallon pots (one crown per pot) under natural sunlight and managed in accordance with conventional practices before subsequent treatments. The culture medium consisted of a 7:2:1 garden soil:peat:perlite (by volume) mixture.

### Two experimental designs and morphological observations for the evaluation of the CR of ‘Hang Baishao’

#### Experiment I: Natural low temperature treatments for CR evaluation

To study the CR of ‘Hang Baishao’ under natural low temperature, the outdoor potted crowns were transferred to a glasshouse (temperature was maintained at 15–25 °C, natural sunlight was used, watering was performed once or twice a week) at intervals of two or 3 weeks from Nov. 26, 2012 to Feb 25, 2013. The seven specific transfer dates are listed in Table 1, and eight natural chilling treatments (always occurring outdoors) were defined as treatments 1–1 to 1–8 (Table 1, referred to as Tre. 1–1 to 1–8, respectively). A total of 21 pots were assigned to each treatment (seven pots per replicate and three replicates per treatment) to observe the performance of sprouting and flowering. All potted crowns were transferred to the glasshouse at 10.00 a.m. on each transfer day.

**Table 1:** Definition and detail of treatments for CR evaluation of ‘Hang Baishao’ under natural and artificial low temperatures, respectively

The date of transferring into glasshouse and/or sampling	Natural low temperature (Experiment I, 2012–2013)	Artificial low temperature (Experiment II, 2013–2014)	Natural low temperature (2015–2016)
Nov. 26	Tre. 1-1^a^		#
Dec. 10	Tre. 1-2^a^		#
Dec. 24	Tre. 1-3^a^		#
Jan. 7	Tre. 1–4		
Jan. 21	Tre. 1-5^a^		#
Feb. 4	Tre. 1-6^a^		#
Feb. 25	Tre. 1-7^a^		#
Outdoors	Tre. 1–8		
Nov. 29		Tre. 2–1	
Dec. 6		Tre. 2–2	
Dec. 13		Tre. 2–3	
Dec. 20		Tre. 2–4	
Dec. 27		Tre. 2–5	
Jan. 3		Tre. 2–6	

^a^Buds were used for RNA-sequencing, and plants were transferred into glasshouse on this day

#Buds were used for qRT-PCR, but plants were not transferred into glasshouse on this day

#### Experiment II: Artificial low temperature treatments for CR evaluation

Five groups of potted crowns were initially placed into refrigerated storage (0–4 °C, cleaned and sterilized beforehand, no light or watering) at 9.00 a.m. on Nov. 29, 2013 and then removed at 9.00 a.m. on Dec. 6, Dec. 13, Dec. 20 and Dec. 27, 2013 and on Jan. 3, 2014; the duration of the chilling treatment lasted for one to 5 weeks. The crowns were then transferred to a glasshouse at 11.00 a.m. on each transfer day (Tre. 2–2 to 2–6, Table 1). Another control group was directly transferred to the glasshouse at 11.00 a.m. on Nov. 29, 2013; this group did not receive any artificial chilling treatment (Tre. 2–1, Table 1). A total of 9 pots were assigned to each treatment (three pots per replicate and three replicates per treatment) to observe sprouting and flowering performance.

#### Morphological observations in experiments I and II

Various morphological indices focusing on sprouting, stem elongation and flowering were measured to provide data for evaluating the CRs of ‘Hang Baishao’ under natural and artificial chilling treatments. The details of these morphological indices and data are shown in Additional files 1, 2, 4, and 5. Both experiments were performed in accordance with a completely randomized design, and analysis of variance (ANOVA) was used to determine the statistical significance of differences in morphological data (Statistical Product and Service Solutions 16.0, SPSS, Chicago, USA).

### CR evaluation on the basis of morphological data

Both chilling hour (CH) and chilling unit (CU) models were adopted on the basis of the morphological data collected in the natural and artificial chilling treatments in order to calculate the CR values of ‘Hang Baishao’. The CR data obtained from experiments I and II were compared and integrated to acquire a reasonable range of CRs for ‘Hang Baishao’.

### Cumulative CH model

The first model is the cumulative CH model. This model defines the CR value as the cumulative number of treatment hours in which the temperature is between 0 and 7.2 °C [21–24]. The temperatures of 0 and 7.2 °C were also included in the calculation of the present study. Natural low temperature values were recorded hourly using ZDR-20 temperature-humidity recorder (Hangzhou Zeda Instruments Limited Company, Hangzhou, CHN).

In experiment I, the start time of the CR calculation was set as 0.00 a.m. on Dec. 8, 2012, during which the daily mean temperature decreased and steadily passed 7.2 °C [21, 22] (Tre. 1–1 was treated as a blank control group that has 0 CHs because its transfer date was Nov. 26, 2012); the end time of each treatment was 10.00 a.m. on each transfer day, at which point each group of potted crowns was transferred to a glasshouse. Because the time interval between the two transfer dates was two or 3 weeks, the calculation end time of Tre. 1–8 plants (always outdoors) was determined to be 2 weeks after the transfer date of Tre. 1–7, namely, 10 a.m. on Mar. 11, 2013.

In experiment II, artificial chilling at any point in time was effective for calculations, because the temperature values were always in the range of 0–4 °C during the treatments in refrigerated storage. This temperature range was within the effective temperature range of 0–7.2 °C. The start time of the calculation was 9.00 a.m. on Nov. 29, 2013, when the five groups of potted crowns were moved into refrigerated storage together, and the end time of each treatment was 9.00 a.m., when each group was removed on its transfer day. The daily mean temperature steadily passed 7.2 °C until Dec. 9, 2013. Before all plants involved in the six treatments were moved into the glasshouse on that day, they had been regarded as accepting any valid natural chilling based on the CH model before they were moved into glasshouse on Nov. 29, 2013; therefore, their subsequent effective chilling accumulations were all derived from artificial chilling during refrigerated storage. The total cumulative chilling on each day was 24 CHs in refrigerated storage, and the durations of the six treatments were 0, 7, 14, 21, 28 and 35 days. Thus, the values of cumulative CHs for the six treatments were six constants, namely, 0, 168, 336, 504, 672 and 840 CHs, respectively.

### Cumulative CU model

The cumulative CU model was also used to evaluate CRs. We referenced this model from herbaceous peony studies of Rhie et al. (2012) and Yeo et al. (2012) in order to compare the CR results among different herbaceous peony cultivars. These authors deduced the linear equation y = −0.0605× + 1 (Eq. 1) based on the results of Fulton [4] and defined y as the value of CU and x as the temperature from 0 to 10 °C (containing 0 and 10 °C in the present study). Additionally, these authors determined that 1 h below 0 °C was equal to 1 CU, whereas 1 h above 10 °C equaled 0 CUs.

In experiment I, the start time of the calculation was defined as 12.00 a.m. on Nov. 30, 2012, as the temperature steadily passed 10 °C on this day (Tre. 1–1 was still the blank control group that had received 0 CUs), and the end time of each treatment was the same as that of the CH model. In experiment II, we did not measure the temperature value per hour in refrigerated storage; therefore, we chose the constant 2 °C, which is in the middle of the range of 0 to 4 °C during refrigerated storage treatments, as the x value of Eq. 1. The start and end time calculations were the same as those in the CH model in experiment II. As with the CH model, six CU values were also six constants: 0, 147.67, 295.34, 443.02, 590.69 and 738.36 CUs.

### Transcriptome sequencing and screening for temperature- and photoperiod-associated genes

#### Sample collection and transcriptome sequencing

**Fig. 2:**
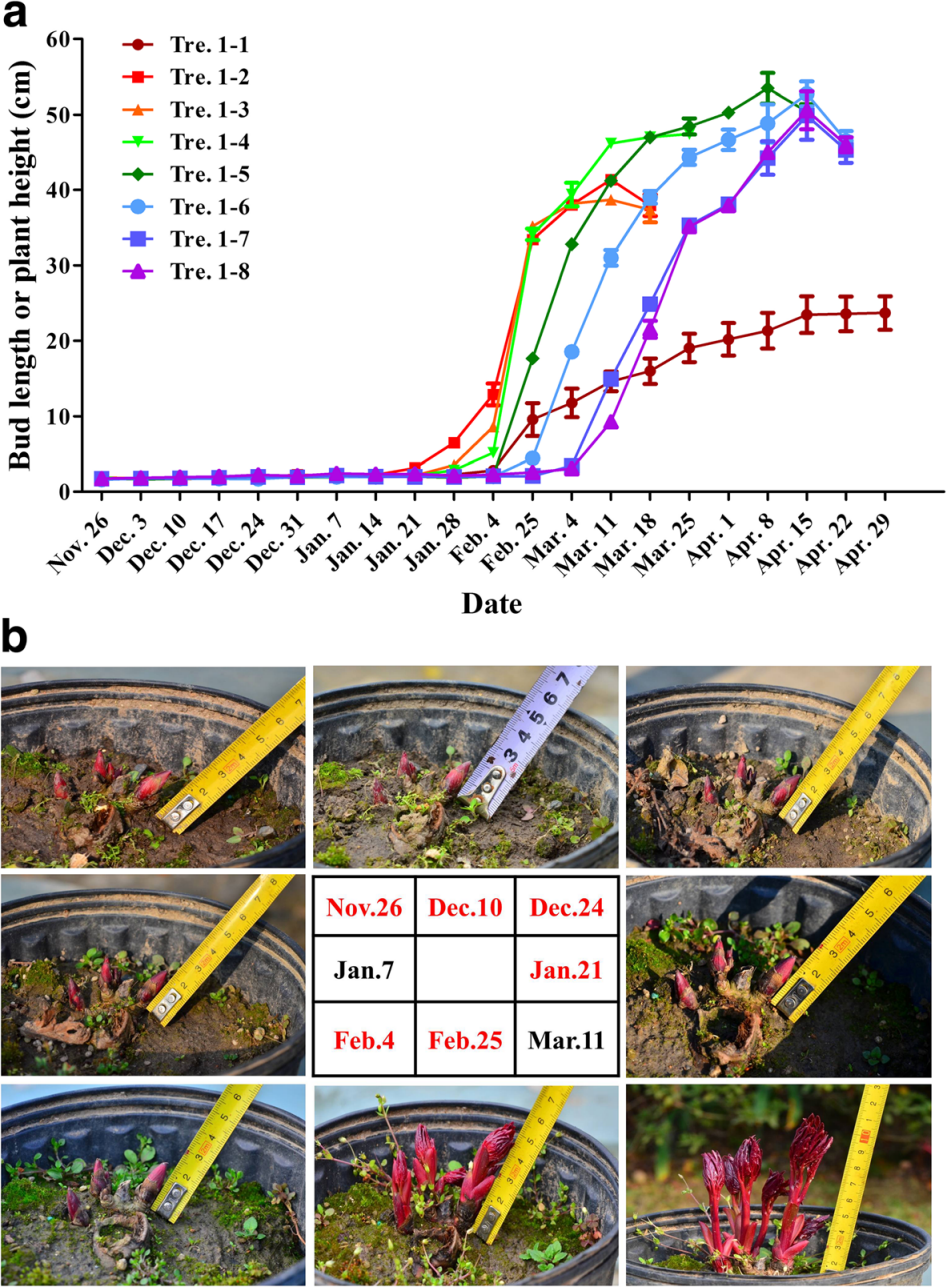
Buds sprouting and growth of ‘Hang Baishao’ under natural low temperature during the entire winter of 2012–2013. **a** Observation on buds sprouting and average plant height in eight treatments; **b** The whole processes of bud dormancy, sprouting and stem growth of a representative plant in Tre. 1–8 which was always stayed outdoor without any artificial heating. Six dates with red color represent six sampling dates for subsequent transcriptome sequencing

The underground buds under natural chilling treatment were sampled in experiment I as materials for transcriptome sequencing. Plump buds (vertical × transverse diameter ≥ 1.5 cm × 0.5 cm, Fig. 2b) on six transfer dates, i.e., Nov. 26, Dec. 10, Dec. 24, 2012 as well as Jan. 21, Feb. 4, and Feb. 25, 2013, were selected and stored at −80 °C after they were flash-frozen in liquid nitrogen. Subsequent morphological analyses (Additional files 1 and 2) showed that the performance of plants from Tre. 1–4 (Jan. 7, 2013) was between that of Tre. 1–3 and Tre. 1–5. Therefore, the samples from Tre. 1–4 were not used for the transcriptome research. After the processes of RNA extraction, cDNA library construction, and deep sequencing using a HiSeq 2000 sequencing platform, six independent gene libraries were constructed and named “Nov. 26”, “Dec. 10”, “Dec. 24”, “Jan. 21”, “Feb. 4”, and “Feb. 25” according to their sampling dates [11].

#### Annotation, screening and expression of genes closely related to temperature and photoperiod responses

Merged and filtered unigene sequences were generated by de novo assembly using “Trinity + Price” and queried via the Basic Local Alignment Search Tool (BLAST) against several other databases, including the NCBI non-redundant protein database (NR), the Swiss-Prot protein database, the Kyoto Encyclopedia of Genes and Genomes (KEGG) database, the Clusters of Orthologous Groups of proteins (COG) database and the Gene Ontology (GO) database. Annotations and descriptions of the putative homologous genes of ‘Hang Baishao’ were mainly obtained from the *Arabidopsis* genome (www.arabidopsis.org).

Various extrinsic and intrinsic factors impact bud dormancy process and CR characteristics, such as water status, hormone content, carbohydrate metabolism, the antioxidase pathway, chromatin remodeling, temperature and photoperiod changes [7, 17, 25–27]. In this study, we mainly focused on genes responding to temperature and photoperiod, which are the two most important ambient factors that directly impact plant dormancy and flowering [28], and ultimately determined the CR amount or fulfillment period.

First, based on these considerations, we preliminarily identified potential differentially expressed genes; second, the unigenes annotated as temperature response, cold response, cold regulated, cold acclimation and deacclimation, etc., or as light harvesting acclimation, light mediated, light chain, photosystem, photosynthesis, phototropin, phytochrome, circadian rhythm, circadian regulation, etc., were further screened from the unigenes expressed differentially. These select temperature- and photoperiod-responsive unigenes were regarded as important candidates for exploring genes that determine the CR characteristics of ‘Hang Baishao’. Gene expression levels were evaluated according to the fragments per kilobase of exon model per million mapped reads (FPKM) value. The expression patterns of these genes were illustrated using a heatmap (Genesis 1.7.6, Institute for Genomics and Bioinformatics, AUT) and analyzed together with morphological data and CR values. We recognize that our RNA-Sequencing data are not replicated, and thus can only serve as an indication of potential genes of interest and a source for sequence data for further investigation.

### Estimation of the CR fulfillment period and qRT-PCR measurements of CR-associated candidate genes during the winter of 2015–2016

#### Estimation of the CR fulfillment period of ‘Hang Baishao’ from November 2015 to February 2016

After the acquiring for the inherent CR of herbaceous peony, we no longer need to depend on cumbersome morphological observations and can use CR values to directly estimate the key period of CR fulfillment for ‘Hang Baishao’ in different years. The CR values of ‘Hang Baishao’ were acquired using the above-mentioned CH and CU models in the winter of 2012–2013. Thus, for the winter of November 2015 to February 2016, the period during which chilling accumulation surpassed the acquired CR values, is the crucial CR fulfillment period of ‘Hang Baishao’ for determining the dormancy release. The CH model was adopted to estimate this key period.

#### Mining of genes possibly involved in the regulation or determination of CR characteristics of ‘hang Baishao’

Based on the former two rounds of screening, 3 years later, we chose 12 representative candidate genes, from the abovementioned select temperature- and photoperiod-responsive unigenes, which expressed actively or changed drastically during several key periods according to the RNA-Seq data. We measured their expression levels in dormant or sprouting buds from November 2015 to February 2016 using qRT-PCR. The six sampling dates in 2015–2016 correspond to those in 2012–2013, which are Nov. 26, Dec. 10 and Dec. 24 of 2015 and Jan. 21, Feb. 4 and Feb. 25 of 2016, respectively (Table 1). The expression patterns of these 12 genes were compared with their RNA-Seq data obtained during the winter of 2012–2013.

#### Primer design, reverse transcription and qRT-PCR tests for select key genes

Corresponding primers were designed based on the sequencing data using Primer Premier 5.0 (Premier Biosoft Inc., Palo Alto, CA) with default parameters for qRT-PCR [29–31]. The unigene021565 annotated as “Ribosomal protein L23/L15e family protein” (RPL15A, AT4G16720) by RNA-Seq showed no significant change in transcriptional levels throughout the whole winter of 2012–2013. Thus, we selected this gene as a reference for normalizing qRT-PCR results. Total RNAs for qRT-PCR were also extracted from buds (Table 1) under natural low temperature using an RNeasy Plant Mini Kit (QIAGEN, 74,904, GER). The cDNA was synthesized from RNA using a PrimeScript™ RT reagent kit with gDNA Eraser (Perfect Real Time) (Takara, RR047A, JPN) in accordance with the manufacturer’s protocol. The genomic DNA was removed in a total volume of 10 μL of reaction mixture that contained 2.0 μL of 5 × gDNA Eraser Buffer, 1.0 μL of gDNA Eraser, 1.0 μg of total RNA and RNase Free dH_2_O, after which the mixture was incubated at 42 °C for 2 min and held at 4 °C. After incubation, 4.0 μL of 5× PrimeScript Buffer 2, 1.0 μL of PrimeScript RT Enzyme Mix I, 1.0 μL of RT Primer, and 4.0 μL of RNase Free dH_2_O were added for reverse transcription at 37 °C for 15 min followed by 85 °C for 5 s, after which the temperature was held at 4 °C [29–32].

The reactions of qRT-PCR were performed using the Platinum® SYBR® Green qPCR SuperMix-UDG with ROX (Invitrogen, C11744–500) with a 20-μL reagent system that contained 10 μL of 2× SYBR® Green (Invitrogen, C11744–500), 1.6 μL of primer mix, 1 μL of RT product and RNase/DNase-free water (AMBION, AM9938). All qRT-PCRs were performed using a Mastercycler® ep Realplex machine (Eppendorf) and carried out under the following conditions: 50 °C for 2 min, 95 °C for 2 min, 95 °C for 15 s, 60 °C for 30 s and 72 °C for 30 s (40 cycles) (melt-curve analysis: 60 °C to 99 °C, 0.5 °C/read). Three technical replicates for each of three biological replicates were performed for each sample. The relative expression levels of these 12 candidate genes were calculated according to the 2^-ΔCt^ method [33, 34].

## Results

### Experiment I: Evaluation of CR under natural low temperature

#### Bud sprouting and growth observations during the entire winter

**Fig. 3:**
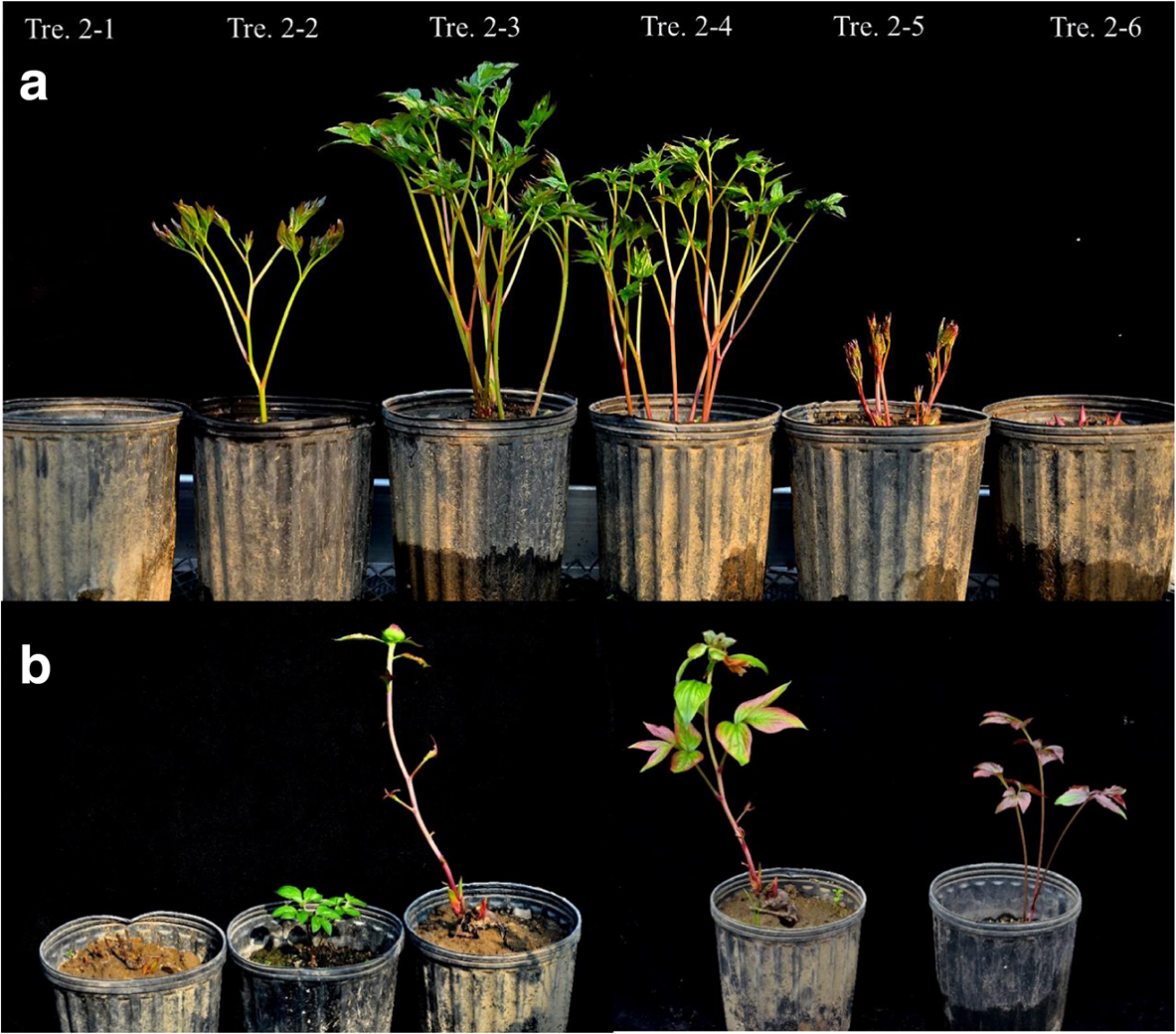
Performances on sprouting and growth of ‘Hang Baishao’ plants under artificial chilling treatments. **a** Growth condition of the representative plants on Jan. 14, 2013 (11 days after the plants of Tre. 2–6 transferred into glasshouse); **b** Several types of abnormal performance on growth and sprouting of the Tre. 2–1 or Tre. 2–2 plants that had serious CR deficiency

The final average plant height of Tre. 1–1 (20–25 cm) was much shorter than that of the others in glasshouse (Fig. 2a, Average Plant Height per plant during the full flowering period (APH) in Additional file 1; observation methods of APH and other morphological indices are elaborated in Additional file 1). Only some plants of Tre. 1–1 sprouted; those that did sprouted very late in the 10 weeks after being transferred to glasshouse (Fig. 2a, average number of Days per replicate between the transfer date and date of stem Elongated visibly for All plants (DEA) in Additional file 1). The stems of these plants grew abnormally and had no flower (Fig. 3b, average Number of Opening Flowers per replicate (NOF) in Additional file 2). All of these negative performances were mainly attributed to the severe shortage of chilling accumulation.

The plant height among Tre. 1–2 to Tre. 1–8 peaked during their respective flowering periods (Period of the Full Flowering (PFF) in Additional file 2). Plant height decreased naturally due to flower withering. Other indices of these eight plant groups concerning sprouting, growth and flowering, such as average Bud Break Percentage per replicate checked 5 weeks after being transferred into glasshouse (BBP), DEA, Average Number of mature and normal Stems per plant during the full flowering period (ANS), average Number of Plants per replicate with Opening flower (NPO), average Number of Opening Flowers per replicate (NOF), average Percentage of Aborted Flowers or per plant (PAF), and PFF (these abbreviations are all elaborated in Additional files 1 and 2), among others, showed clear gradient changes (Tables 1 and 2). One of the Tre. 1–8 plants was seen as a specimen for natural dormancy and sprouting observations of ‘Hang Baishao’ (Fig. 2b).

**Table 2:** Chilling accumulation of ‘Hang Baishao’ plants under natural and artificial low temperature

Treatment	Transfer date	CH (h)	CU	Treatment	Transfer date	CH (h)	CU
Tre. 1–1	Nov. 26, 2012	0.00	0.00	Tre. 2–1	Nov. 29, 2013	0.00	0.00
Tre. 1–2	Dec. 10, 2012	46.00	117.47	Tre. 2–2	Dec. 6, 2013	168.00	147.67
Tre. 1–3	Dec. 24, 2012	250.00	334.84	Tre. 2–3	Dec. 13, 2013	336.00	295.34
Tre. 1–4	Jan. 7, 2013	483.00	615.27	Tre. 2–4	Dec. 20, 2013	504.00	443.02
Tre. 1–5	Jan. 21, 2013	738.00	856.08	Tre. 2–5	Dec. 27, 2013	672.00	590.69
Tre. 1–6	Feb. 4, 2013	892.00	1009.45	Tre. 2–6	Jan. 3, 2014	840.00	738.36
Tre. 1–7	Feb. 25, 2013	1170.00	1311.58				
Tre. 1–8	Mar. 11, 2013	1238.00	1383.20				

#### Evaluation of CR for ‘Hang Baishao’ under natural low temperature

To determine the CR of ‘Hang Baishao’ required to break bud dormancy and achieve full bloom, we measured various morphological indices and calculated natural cumulative chilling values (Additional files 1 and 2). These data indicated that chilling significantly influenced the percentage of ‘Hang Baishao’ budbreak and blossoms. Chilling accumulation increased as the transfer date was delayed. The values of BBP, average Final Bud break Percentage per replicate after being transferred into glasshouse (FBP), ANS, APH, Average Plant Width per plant during the full flowering period (APW) (Additional file 1), NPO, NOF, Average Number of Opening and Aborted flowers per plant (ANO and ANA), average Percentage of Opening Flowers per plant (POF), Average Diameter of Flowers per flower (ADF) and Average Diameter of flower Stems per flower (ADS) (Additional file 2) increased to different degrees under continuous chilling accumulation; whereas DEA (Additional file 1), PAF and Number of the sustaining Days of Full flowering (NDF) (Additional file 2) gradually decreased as the transfer date was delayed. The values of the average Number of Small bud-Points and Abnormal Buds per plant (NSP and NAB) (Additional file 1) and Average Opening Days per flower (AOD) (Additional file 2) changed irregularly.

Daily temperatures were tested hourly from Nov. 23, 2012 to Mar. 11, 2013, as shown in Additional file 3. The partial buds of Tre. 1–1 diminished and never sprouted (71%, FBP in Additional file 1); these plants did not receive any effective accumulation of CHs or CUs before being transferred to the glasshouse on Nov. 26, 2012. In response to increased chilling and cultivation in the glasshouse for 5 weeks [35], buds partially sprouted (19–62%, BBP in Additional file 1) in plants that received approximately 46.00 to 250.00 CHs or 117.47 to 334.84 CUs (Tre. 1–2 to 1–3). These plants blossomed with fewer ANS, APH, APW (Additional file 1), NPO, NOF, ANO and ADF compared with those of the last four treatments (Additional file 2). After the chilling treatment of 483 .00 CHs or 615.27 CUs, several important growth and flowering indices of Tre. 1–4 plants, including BBP, ANS, APH, APW (Additional file 1), NPO, NOF and ANO (Additional file 2), significantly increased compared with those of Tre. 1–3 plants. The main growth and flowering performances of Tre. 1–5 were similar to those of Tre. 1–4, whereas three important indices, i.e., DEA (Additional file 1), ANO and ADS (Additional file 2), were significantly better than those of Tre. 1–4.

Plants of Tre. 1–6 to 1–8 were subjected to a prolonged low temperature treatment that exceeded the 738.00 CHs and 856.08 CUs of Tre. 1–5 plants, but most morphological differences among Tre. 1–6, 1–7 and 1–8 plants were not distinct (Fig. 2a, Additional files 1 and 2). The data showed that the optimal CR was 738.00 CHs or 856.08 CUs for ‘Hang Baishao’ to achieve its comprehensive best performance of sprouting, growth and flowering.

### Experiment II: evaluation of CR under artificial low temperature

As shown in Additional files 4 and 5, artificial low temperature also clearly promoted the sprouting and flowering of ‘Hang Baishao’. The values of ANS, APH, APW, average Relative Content of Chlorophyll of leaves (RCC) (Additional file 4), NPO, average Percentage of Plants with Opening flower per replicate (PPO), NOF, ANO and ANA (Additional file 5) displayed an overall increasing tendency as artificial chilling persisted and as the transfer date was delayed; whereas average number of Days per repliacate between the transfer date and date of the First plant Sprouting (DFS) (Additional file 4) or Flowering (DFF) and POF (Additional file 5) mostly decreased gradually. The values of FBP, Average Leaf Length, Width and Thickness per plant during the full flowering period (ALL, ALW, ALT, Additional file 4), AOD and ADF (Additional file 5) changed erratically. Figure 3a shows the performances of budbreak and growth of six representative plants on the 11th day after the last group (Tre. 2–6) was transferred to the glasshouse. The plants of Tre. 2–1 and 2–2 acquired severely inadequate chilling accumulation, which manifested as several abnormal performances in growth, such as no sprouting, very limited plant height and width, only one large and malformed flower bud on the top of plants that lacked normal leaves, only one delicate stem, and leaf surfaces and stems that presented morbid color or odd stripes (Fig. 3b). These abnormalities also occurred in the plants that acquired inadequate chilling accumulations in experiment I.

After comprehensive comparison, the results showed that the growth and flowering performances of Tre. 2–5 plants (672.00 CHs or 590.69 CUs) were best with respect to many important indices, such as the maximum values of FBP, ADS, APH, APW (Additional file 4), NPO, PPO, NOF and ANO (Additional file 5); Tre. 2–5 plants also exhibited larger values of ANS (Additional file 4), minimum values of DFS (Additional file 4) and smaller values of DFF (Additional file 5). Tre. 2–6 plants might have suffered excessive chilling (840.00 CHs or 738.36 CUs) that resulted in poorer performance of growth and flowering compared with Tre. 2–5. For example, the average number of flowers per plant of Tre. 2–6 (ANO, only 1.00 in Additional file 5) was significantly less than that of Tre. 2–5 (2.33 in Additional file 5), whereas the DFS (14.33 in Additional file 4) of Tre. 2–6 was greater than that of Tre. 2–5 (11.00 in Additional file 4). Therefore, a total of 672.00 CHs or 590.69 CUs for Tre. 2–5 plants was the optimal chilling accumulation for ‘Hang Baishao’ to break bud dormancy, grow vigorously and bloom exuberantly based on the chilling treatment by refrigerated storage.

### Comparison of CR values obtained from natural and artificial chilling treatments

The chilling accumulations of ‘Hang Baishao’ obtained in experiments I and II are listed in Table 2. The optimal CRs of ‘Hang Baishao’ were 738.00 CHs or 856.08 CUs under natural chilling treatment and 672.00 CHs or 590.69 CUs under artificial low temperature.

The lowest CR value (threshold) of ‘Hang Baishao’ was between 483.00 and 738.00 CHs or between 615.27 and 856.08 CUs under natural chilling conditions (between the chilling accumulations of Tre. 1–4 and 1–5). Interestingly, the interval of 504.00 to 672.00 CHs obtained from artificial chilling is just enclosed in the range of 483.00 to 738.00 CHs (Table 2). Therefore, the lowest CR of ‘Hang Baishao’ should be between 504.00 and 672.00 CHs from the intersection of these two ranges. On the other hand, for the CU model, there was no intersection between the two intervals of 615.27 to 856.08 CUs and 443.02 to 590.69 CUs (Table 2).

### Summary of transcriptome sequencing and gene assembly

The buds were collected at six selected date of winter (Fig. 2b) were assigned to six independent gene pools, which were marked as Nov. 26, Dec. 10, Dec. 24, Jan. 21, Feb. 4 and Feb. 25. A total of 294,792,058 raw reads, 286,910,694 clean reads, 294,792,058,000 nucleotides and 51,481 non-redundant unigenes with a mean length of 1250 bp were obtained in all six pools by the assembly method of “Trinity + PRICE”. The maximum and minimum bases of the unigenes were 14,624 and 200, respectively, and the N50 and N90 lengths were 1804 and 597, respectively. The clean sequencing data were submitted to the Sequence Read Archive (SRA) of NCBI, and the assembled unigenes were uploaded to the Transcriptome Shotgun Assembly (TSA) database at NCBI. The available BioProject accession number is PRJNA245064. The TSA project was deposited in the DNA Data Bank of Japan (DDBJ)/European Molecular Biology Laboratory (EMBL)/GenBank databases under accession GBFN00000000. The SRA run accessions (SRR) of the clean sequencing data derived from the six gene libraries of Nov. 26, Dec. 10, Dec. 24, Jan. 21, Feb. 4, and Feb. 25 are SRR1258112, SRR1258117, SRR1269644, SRR1269649, SRR1269650 and SRR1269651, respectively. A total of 2057 genes with large differences in accumulation between samples were identified and used both as screening objects for the genes closely linked with temperature and photoperiod factors and to determine the CR characteristics of ‘Hang Baishao’. Detailed information concerning the transcriptome sequencing was shown and analyzed in our previous publication and therefore does not need to be repeated here [11].

### Screening of genes that likely respond to temperature and photoperiod

A total of 66 unigenes with substantial differences in accumulation between samples screened from the differentially expressed unigenes were annotated as temperature- and photoperiod-associated genes. These unigenes could be classified into 33 types in reference to the *Arabidopsis* genome (Table 3). Among these unigenes, the majority were directly annotated as heat shock responsive (17 in total, i.e., *HSP*, *HSC* and *HSF* in Table 3); the unigenes annotated as *BIP1*, *ERD2*, and Unigene006523 (*Arabidopsis* gene name is unknown) were also indirectly concerned with heat shock according to the information of the *Arabidopsis* description in Additional file 6. The unigenes with the annotation of *OSM* (6), *LOS1* (5) and *COR47* (4) are also clearly more abundant than the others. Genes with minimal expression differences between samples but with similarity to genes previously shown to be involved in the temperature and photoperiod response were also identified and selected (Additional file 7), as these genes are also possible candidates for promoting bud dormancy and directly determining CR characteristics.

**Table 3:** Abbreviation and number of the unigenes representing homologues most similar to *Arabidopsis* genome mentioned in the present manuscript

Abbreviation	Full name	Number
*HSP*	*HEAT SHOCK PROTEIN*	17
*HSC*	*HEAT SHOCK COGNATE PROTEIN*	
*HSF*	*HEAT SHOCK TRANSCRIPTION FACTOR*	
*BIP1*	*BINDING PROTEIN*	1
*ERD2*	*EARLY-RESPONSIVE TO DEHYDRATION 2*	1
*OSM*	*OSMOTIN*	6
*LOS1*	*LOW EXPRESSION OF OSMOTICALLY RESPONSIVE GENES 1*	5
*AGL20*	*AGAMOUS-LIKE 20*	2
*SOC1*	*SUPPRESSPOR OF OVEREXPRESSION OF CONSTANS1*	
*COR47*	*COLD-REGULATED 47*	4
*WRKY33*	*WRKY DNA-BINDING PROTEIN 33*	1
*ATDGK2*	*DIACYLGLYCEROL KINASE 2*	1
*ATPP2CA*	*PROTEIN PHOSPHATASE 2CA*	1
*GAPB*	*GLYCERALDEHYDE-3-PHOSPHATE DEHYDROGENASE B SUBUNIT*	1
*LTI30*	*LOW TEMPERATURE-INDUCED 30*	1
*RAB18*	*RESPONSIVE TO ABA 18*	1
*RCI2B*	*RARE-COLD-INDUCIBLE 2B*	1
*SEX1*	*STARCH EXCESS 1*	1
*TCH4*	*TOUCH 4*	2
*PAP2*	*PHYTOCHROME-ASSOCIATED PROTEIN 2*	1
*LHCB4.2*	*LIGHT HARVESTING COMPLEX PHOTOSYSTEM II*	1
*PGK*	*PHOSPHOGLYCERATE KINASE*	1
*ATNADP-ME3*	*NADP-MALIC ENZYME 3*	1
*CAM8*	*CALMODULIN 8*	1
*F11 M15.26*		1
*LHCA6*	*PHOTOSYSTEM I LIGHT HARVESTING COMPLEX GENE 6*	1
*PCK1*	*PHOSPHOENOLPYRUVATE CARBOXYKINASE 1*	2
*PCK2*	*PHOSPHOENOLPYRUVATE CARBOXYKINASE 2*	1
*PSBP-1*	*PHOTOSYSTEM II SUBUNIT P-1*	1
*SBPASE*	*SEDOHEPTULOSE-BISPHOSPHATASE*	2
*LHY*	*LATE ELONGATED HYPOCOTYL*	1
*TOC1*	*TIMEING OF CAB EXPRESSION 1*	1
*APRR3*	*PSEUDO-RESPONSE REGULATOR 3*	2
No Arabidopsis gene name		3
Total		66

### Expression patterns of temperature- and photoperiod-associated unigenes under natural low winter temperatures from November 2012 to February 2013

**Fig. 4:**
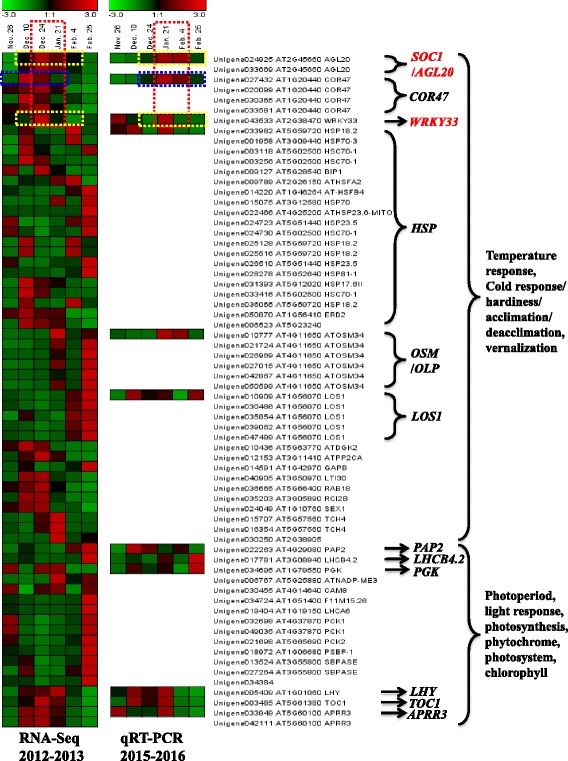
Heatmap diagrams of the differentially expressed unigenes related to ambient temperature and photoperiod. The two heatmaps are used to show the relative expression trends of key candidate genes which were probably associated with temperature or photoperiod changes, and possibly involved in impacting CR characteristic of ‘Hang Baishao’. Red cell means up-regulation and green cell means down-regulation. Bars on the top indicate the range of gene expression change, values (−3 to +3) of which were normalized from FPKMs value of unigene by using Genesis 1.7.6. On the right side of two heatmap diagrams, the first column is Gene ID, the second is *Arabidopsis* homology, and the third one is gene name of *Arabidopsis*. Abbreviations of partial important unigenes, and their closely related factors or processes were labeled on the right side of these three columns. The expression patterns of Unigene003256, Unigene050870, Unigene022263 and Unigene005409 based on RNA-Seq data had been analyzed in our previous publication [11]. We added the expression data of these four genes here again in order to facilitate the relevant presentation and discussion in the current study, and also facilitate the comparative analysis of RNA-Seq data of 2012–2013 and qRT-PCR data of 2015–2016 for Unigene022263 and Unigene005409

On the basis of the data of our previous publication, we divided the period from Nov. 26, 2012 to Feb. 25, 2013 into five phases: (1) Nov. 26 (Tre. 1–1) and before this date, bud endodormancy; (2) Nov. 26 (Tre. 1–1) to Dec. 10 (Tre. 1–2), bud dormancy transition; (3) Dec. 10 (Tre. 1–2) to Jan. 21 (Tre. 1–5), ecodormancy; (4) Jan. 21 (Tre. 1–5) to Feb. 4 (Tre. 1–6), ecodormancy release; (5) after Feb. 4 (Tre. 1–6), bud sprouting and elongation (Fig. 6) [11, 36]. After combining the results of the dormancy phases, the heatmap in Fig. 4 and the annotation information in Additional file 6, we hypothesize that the unigenes annotated as *SOC1*, *COR47*, *WRKY33*, *LHY*, *TOC1* and *APRR3* are actively expressed during the ecodormancy period from Dec. 10 to Jan. 21 on the basis of RNA-Seq data. *HSP* genes had comparatively high FPKM values from Dec. 10 to Dec. 24 and from Feb. 4 to Feb. 25. The expression levels of the *OSM* and *LOS1* genes and most photoperiod-responsive genes, i.e., *PAP2*, *LHCB4.2*, *PCK1* and *PCK2*, sharply increased during the last period of Feb. 25 (Fig. 4). In addition, there were relatively fewer highly expressed genes in the endodormancy phase of Nov. 26 compared with the other phases.

### Estimation of the CR fulfillment period for dormancy release from November 2015 to February 2016

**Fig. 5:**
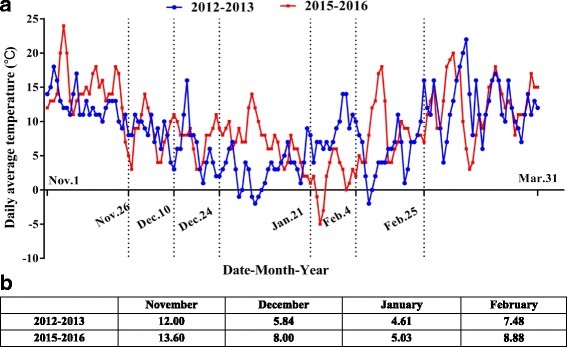
Comparison of natural low temperatures between the two winters. **a** Temperature changes from Nov. 1 to Mar. 31 of 2012–2013 and 2015–2016; **b** Comparison of mean monthly temperatures between these two winters

The comparison of temperature changes and mean monthly temperatures between the winters of 2012–2013 and 2015–2016 are shown in Fig. 5. The temperature records from November 2015 to March 2016 are listed in Additional file 8. After combining the data from Fig. 5 and Additional files 3 and 8, we could find that the temperatures during the four winter months of 2015–2016 were overall clearly higher than those of 2012–2013.

Table 4 shows the accumulation of CHs during both winters. In accordance with the previous results, if ‘Hang Baishao’ acquires more than 672.00 CHs, it would gradually break bud dormancy and grow vigorously (Table 2). Therefore, the time interval of ‘Hang Baishao’ to acquire sufficient CHs during the winter of 2015–2016 was between Jan. 21 and Feb. 4 of 2016 (Table 4, 857 CHs in Feb. 4, which is greater than 672 CHs), which occurred approximately 2 weeks later than that in 2012–2013. Hence, the key period of CR fulfillment was delayed. This phenomenon is consistent with the greater overall temperatures during the winter of 2015–2016 compared with those of 2012–2013 (Fig. 5).

**Table 4:** Comparison of accumulated CHs between two winters and estimation for the key period of ‘Hang Baishao’ CR fulfillment in the winter of 2015 to 2016

Accumulated CHs in each time interval			Accumulated CHs in each key time point		
	2012–2013	2015–2016		2012–2013	2015–2016
Before Nov. 26	0	33	Nov. 26	0	33
Nov. 26-Dec. 10	103	128	Dec. 10	103	161
Dec. 10-Dec. 24	204	107	Dec. 24	307	268
Dec. 24-Jan. 21	488	331	Jan. 21	795^a^	599
Jan. 21-Feb. 4	154	258	Feb. 4	949	857^a^
Feb. 4-Feb. 25	278	227	Feb. 25	1227	1084

^a^The period in that year when ‘Hang Baishao’ acquired sufficient CR. The period of CR fulfillment of ‘Hang Baishao’ in 2015–2016 was delayed for 2 weeks comparing with that in 2012–2013

### qRT-PCR measurements of 12 select genes relevant to temperature and photoperiod responses during the winter of 2015–2016

Three years later, twelve unigenes were further screened from the 66 abovementioned temperature- and photoperiod-responsive unigenes (Table 3). The expression levels of these unigenes in dormant or sprouting buds collected in 2015–2016 were measured again using qRT-PCR in order to detect differences in their expression between two winters 3 years apart (Fig. 4), and to test the hypothesis that expression of these genes correlated with the CR fulfillment of ‘Hang Baishao’. The primer sequences of these 12 genes are shown in Table 5.

**Table 5:** Primer sequences of 12 representative genes which were relevant to environment temperature and photoperiod

Unigene information and annotation	Primer sequence
Unigene033982, AT5G59720, *HSP18.2*	F^a^: GGCTTCTTCTCCTCCTGTTTAG
	R^a^: GTTCAGGTTGCCGGAGAAT
Unigene010777, AT4G11650, *ATOSM34*	F: CCGTAACAGGGCTCAAATCTAT
	R: CATCCATTACTCTAGCCGAGTTC
Unigene010909, AT1G56070, *LOS1*	F: CAACCATGGCAACTGTGTTAC
	R: GGCGAGAAGAAGGATCTGTATG
Unigene024925, AT2G45660, *AGL20*	F: CGGCAGCGATGAAATAGTAGA
	R: ATCCCAAACCTTCTCCCATTAG
Unigene027432, AT1G20440, *COR47*	F: CGGCAGCGATGAAATAGTAGA
	R: ATCCCAAACCTTCTCCCATTAG
Unigene043533, AT2G38470, *WRKY33*	F: ACGCCTGAGAATTCGTCATTAT
	R: TTGGGCTCAGGTTCATCTTC
Unigene022263, AT4G29080, *PAP2*	F: AAAGGTCTGAGCTGCCATATC
	R: ACTTATGATCCACAGCCTCTTAC
Unigene017781, AT3G08940, *LHCB4.2*	F: GGTCCAAGGAGTCGTAATCAAA
	R: CGAATGGCTCGATGGAAGTAT
Unigene034695, AT1G79550, *PGK*	F: GGAATTGTGATGGCGACAAAG
	R: TCTTCTGGTTATCGTCCAAAGG
Unigene005409, AT1G01060, *LHY*	F: GTGCAGGCTTTCGAGGATAA
	R: GAGAAGGAGGCTACGGTTAATG
Unigene003485 AT5G61380, *TOC1*	F: TTCTCGGATGACACAGATGATAAG
	R: TAGTAGCAGCAGCAGCATTAG
Unigene033949, AT5G60100, *APRR3*	F: CCAGATAAAGAGGGCATGACTAC
	R: GGTTGTTGCTGTTGCAGATG

^a^ *F* Forward Prime, *R* Reverse Prime

The expression patterns of the 12 genes in 2015–2016 exhibited clear differences to different degrees compared with those in 2012–2013 (Fig. 4). Three types of congruent relationships regarding expression patterns obtained using RNA-Seq and qRT-PCR in both winters were observed and are summarized in Table 6. The differences in gene expression patterns between the two winters and the related reasons or mechanisms are analyzed in detail in the following discussion section.

**Table 6:** Three types of congruent relationship of 12 gene expression patterns obtained from qRT-PCR in 2015–2016 and RNA-Seq in 2012–2013

Type	Gene	Description
Similar overall, but possibly advanced or delayed	Unigene024925 (*SOC1*/*AGL20*) Unigene027432 (*COR47*) Unigene043533 (*WRKY33*)	The periods of gene up- or down-regulated obviously in 2015–16 seem like to be advanced or delayed for several weeks comparing with those in 2012–13
Partially similar	Unigene033982 (*HSP18.2*) Unigene010777 (*ATOSM34*) Unigene017781 (*LHCB4.2*) Unigene005409 (*LHY*) Unigene003485 (*TOC1*)	The periods of gene highly expressed, or up- or down-regulated obviously in 2015–16 are partially similar with those in 2012–13
Totally different, irregular	Unigene010909 (*LOS1*) Unigene022263 (*PAP2*) Unigene034695 (*PGK*) Unigene033949 (*APRR3*)	Fluctuant and irregular expression patterns, hardly any similarity between two winters

## Discussion

### CR results obtained from two types of chilling treatment are reliable and practical

The CH model has been frequently used in many studies on deciduous fruit trees [8, 23]. We designed two experiments in continuous 2 years to evaluate the CRs of ‘Hang Baishao’ and ultimately identified the intersection of the CH amount according to the results from natural and artificial chilling treatments. The results based on the two different chilling experiments are more reliable than those obtained only from one of natural or artificial chilling treatments.

The lowest CR of ‘Hang Baishao’ was between 504.00 and 672.00 CHs to ensure good growth and flowering. If the cumulative chilling amount of ‘Hang Baishao’ was closer to 672.00 CHs rather than 504.00 CHs, the comprehensive performance of sprouting, growth and flowering was better (Additional files 1, 2, 3, and 4). Therefore, the optimal CR amount of ‘Hang Baishao’ is 672.00 CHs based on the combined results from both experiments. If planters want to ensure ‘Hang Baishao’ breaks bud dormancy, sprouts and blooms sufficiently, planters must make sure that ‘Hang Baishao’ plants acquire chilling amounts equal to or greater than 672.00 CHs. Our analyses of the CR interval and the lowest and optimal CR amounts are similar to those of a study on CR fulfillment and related transcript profiling of *Vitis riparia* [8], and have been confirmed to be feasible and practical for CR research on herbaceous peony.

### ‘Hang Baishao’ is likely a relatively low-CR germplasm of *P. lactiflora* and deserves enough attention and further study

**Fig. 6:**
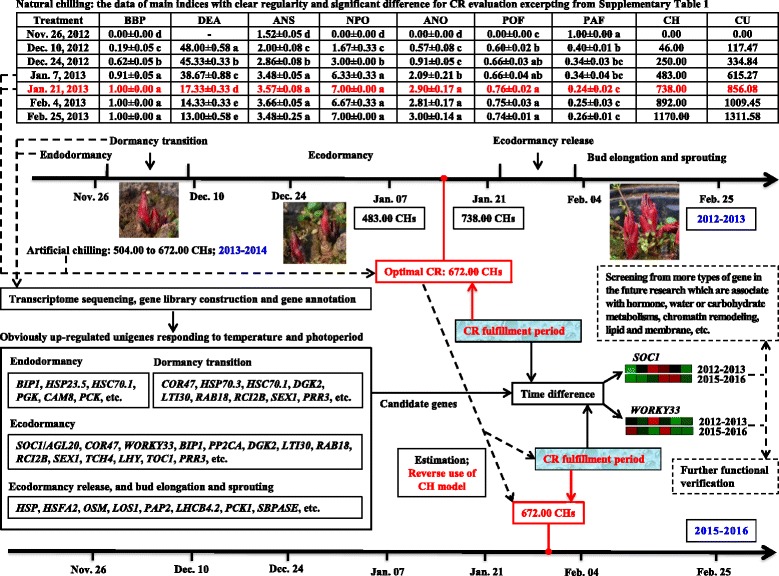
The research framework and main results of this study

Regrettably, the CH results could not be compared with those obtained from other *P. lactiflora* cultivars because this model has not been previously used in the study of herbaceous peony. In the present study, the CH model was mainly used to evaluate the CR value and estimate the CR fulfillment period in the winter of 2015–2016, which constitutes a “reverse use” of the CH model (Fig. 6, Table 4); whereas the CU model was adopted in order to compare the CR results with those of other herbaceous peony germplasms.

Based on an identical evaluation system, Yeo et al. (2012) reported that 1222 CUs was a recommended natural chilling amount for breaking dormancy and flowering of *P. lactiflora* ‘Taebaek’ [10]; similarly, Rhie et al. (2012) reported that 1058 CUs was an adequate artificial chilling amount for dormancy release and normal growth of ‘Taebaek’ and ‘Mulsurae’ [9]. However, ‘Hang Baishao’ only needed 856.08 CUs and 590.69 CUs in two chilling treatments to both enable 100% of plants to break bud dormancy (FBP of both Tre. 1–5 in Additional file 1 and Tre. 2–5 in Additional file 4) and produce more stems (ANS in Additional files 1 and 4) and flowers (ANO in Additional files 2 and 5).

Rhie et al. (2012) also reported that the numbers of CUs required for 95% of ‘Taebaek’ and ‘Mulsurae’ plants to sprout were 763 and 913 CUs, respectively. In the present study, only 615.27 CUs were needed for 91% of the crowns of ‘Hang Baishao’ to sprout (BBP of Tre. 1–4 in Additional file 1). Furthermore, the CUs required to reach 95% of the potential number of stems per plant (approximately three in the study of Rhie et al. (2012)) were 1078 and 1323 in ‘Taebaek’ and ‘Mulsurae’, respectively. However, ‘Hang Baishao’ could produce more than three stems per plant (ANS of both Tre. 1–4 in Additional file 1 and Tre. 2–3 in Additional file 4) by receiving only 615.27 natural CUs or 295.34 artificial CUs. Our results confirmed that ‘Hang Baishao’ has a significantly lower CR.

At present, only two cultivars have been studied using the CU model in addition to ‘Hang Baishao’; therefore, the CU amount of more cultivars needs to be evaluated to confirm the comparatively lower CR of ‘Hang Baishao’. In another aspect, the hypothesis that ‘Hang Baishao’ is probably a low-CR germplasm of *P. lactiflora* has also been proven for many years by our cultivation experiences. We have introduced more than 30 ornamental *P. lactiflora* cultivars that have double or more complex flower types from Heze City (E 114°48′-116°24′, N 34°39′-35°52′), which is located in the northern part of China and has a relatively cooler climate compared with Hangzhou. We planted ‘Hang Baishao’ in the same resource nursery as Heze cultivars. After systematic observations for 5 years, many Heze cultivars showed uneven budbreak and clearly fewer flowers, and these cultivars grew weaker each year and even died. In contrast, ‘Hang Baishao’ always grew vigorously and blossomed early and well in the spring during the past 5 years. The low CR characteristics of ‘Hang Baishao’ was likely due to its long cultivation history of more than 1800 years in Zhejiang. This germplasm should receive more attention as an important material in garden, landscape and cut-flower applications in the subtropical areas of the Northern Hemisphere, and also as a pioneer breeding parent for acquiring superior cultivars that have strong adaptability under warm winters and springs.

### Temperature- and photoperiod-associated genes are the main putative candidates for exploring genes that regulate or determine CR characteristics

Many different genes are involved in the regulation of different metabolic or signal transduction pathways during plant dormancy and flowering [13, 14, 26, 37]. The biological function and expression patterns of these genes can likely determine the time or duration of dormancy transition, release and sprouting [8, 17, 38], in other words, determine the CR amount or fulfillment period. As mentioned above, the CR is an inherent attribute for plants breaking dormancy and promoting budbreak and is regulated by multiple factors, including changes in temperature and daylight, the interconversion of free and bound water, the contents of endogenous hormones and carbohydrates, chromatin remodeling, lipids and membranes, and the activity of antioxidant enzymes [7, 11–14, 25, 27, 39–41]. Temperature and photoperiod are the two most important natural factors that directly advance or delay the dormancy process; therefore, genes that respond to or are associated with temperature and photoperiod certainly play crucial roles during bud dormancy transition and release [12, 13] and are major candidates for exploring genes that are closely related to or directly determine the CR characteristics of herbaceous peony.

The *HSP*, *OSM*, *AGL*, *LOS1*, *SOC1*, *AGL* and *COR47* genes, among others, are known for their sensitivity to temperature changes [7, 12, 42, 43]; on the other hand, genes annotated as *PAP2*, *LHCB4.2*, *PGK*, *LHY*, *TOC1* and *APRR3* are related to photoperiod/daylight or participate in circadian rhythm/clock processes [7, 25]. These genes showed different expression patterns during the dormancy and sprouting of ‘Hang Baishao’, which can reveal much information about gene regulation during dormancy release and CR fulfillment.

### *SOC1* and *WRKY33* are likely involved in the direct determination of the CR characteristics of ‘hang Baishao’

#### *SOC1* and *AGL20* are representative temperature-responsive genes and flowering integrators

Unigene024925 and Unigene033669 were annotated as homologs of *AGAMOUS LIKE 20* (*AGL20*) according to the *Arabidopsis* genome. *AGL20* and its homologous *AGL24* genes have been proved to be flora promoters and are associated with the *DORMANCY-ASSOCIATED MADS-box* (*DAM*) gene. These genes respond to cold temperature and short photoperiods and regulate both the dormancy induction and release in leafy spurge, pear and poplar [12, 15, 42–44]. *SOC1* is another gene name of *AGL20*, [45] which is a temperature-responsive gene and flowering integrator that is active in the shoot apical meristem, growing leaves and underground bulbs [38, 42, 46]. *SOC1*/*AGL20* regulates floral patterning and flowering time by integrating various flowering pathways derived from temperature, photoperiod and hormones [37, 46–49]. Vernalization-induced *SOC1* and its repressor *FLOWERING LOCUS T* (*FT*) can activate the genes involved in floral meristem identity, i.e., *LEAFY* (*LFY*) and *APETALA 1* (*AP1*), ultimately accelerating floral initiation [20, 38, 42, 46, 50]. In addition, the transcriptional patterns of pear *SOC1* are similar to those of *DAM* genes, which could indicate that *SOC1* might also play a crucial role in controlling the process of dormancy transition and release [15]. In general, *SOC1* genes are closely associated with bud dormancy and flowering. Understanding the potential connection between *SOC1* and CR is important for determining if it is a key gene in dormancy transitions.

#### Allelic variation of *SOC1* and the CR amount/fulfillment period

Studies on the relationship between *SOC1* expression or regulation and CR fulfillment are rare; only Trainin et al. (2013) have reported interesting results. They confirmed that *ParSOC1* expression was closely linked with the CR for bud dormancy release in apricot (*Prunus armeniaca* L). Interestingly, apricot cultivars with different CRs had different allelic combinations of *ParSOC1*: high-CR apricot cultivars had 262/262 and 262/278 allelic combinations, whereas low-CR cultivars had 215/278 allelic combinations. This study did not reveal the immediate cause of the congruent relationship between the CR amount and allele combination of *SOC1*, and did not confirm that allelic variation of *ParSOC1* was the sole decisive factor that determines CR differences among different apricot cultivars [15]. However, these innovative and valuable results have already received attention by many other researchers [45, 51] and have provided a reference for the future screening and deep mining of genes that underlie plant CRs [52].

#### *SOC1* and *WRKY33* likely participate in the determination of the CR characteristics of ‘Hang Baishao’

In the present study, two *SOC1* genes showed relatively high expression levels between Dec. 24, 2012 and Jan. 21, 2013, which is the key period for the CR fulfillment of ‘Hang Baishao’ on the basis of the RNA-Seq data (red box with dotted line in Fig. 4, Table 2, Additional files 1, 2). These two genes were gradually up-regulated before Dec. 24 (insufficient CR) but rapidly down-regulated after Jan. 21 (sufficient CR). Thus, a certain relationship may exist between the active expression of *SOC1* and the CR fulfillment of ‘Hang Baishao’.

On the basis of the qRT-PCR data obtained from 2015 to 2016, the period of comparatively high expression and sharp down-regulation of Unigene024925 (one of two *SOC1* genes) was delayed for two to 3 weeks in 2015–2016 compared with that in 2012–2013 (yellow box with dotted line in Fig. 4). Interestingly, this time delay of active *SOC1* expression is consistent with the delay of the CR fulfillment period in 2015–2016 (Fig. 6, Jan. 21 → Feb. 4 with symbol “*” in Table 4). This phenomenon further confirmed the strong positive correlation between *SOC1* expression and the sufficient CR fulfillment of ‘Hang Baishao’. Therefore, *SOC1* likely participates in the regulation or determination of the CR amount and fulfillment period of ‘Hang Baishao’.

The same phenomenon also occurred for Unigene043533 (*WRKY33*), which is also illustrated by two yellow boxes with dotted line in Fig. 4. Both *SOC1* and *WRKY33* are functionally conserved genes with potential roles in breaking dormancy and promoting flowering [19, 26]. Regarding Unigene027432 (*COR47*), its periods of high expression and clear down-regulation were also delayed, but the delay persisted for more than 1 month (two blue boxes with dotted line in Fig. 4). Therefore, this delay is too long to be consistent with the delay of the CR fulfillment period. *COR47* and *WRKY33* respond to sugar starvation, dehydration and other abiotic stresses and may regulate the processes of ecodormancy, cold response and acclimation [15, 16, 53].

The internal mechanism of *SOC1* and *WRKY33* affect the CR fulfillment, and the potential relationship between the allelic combinations of *SOC1* or *WRKY33* and the CR amount or fulfillment period of ‘Hang Baishao’ are still unknown in our current research. In summary, *SOC1* and *WRKY33* are two potential candidate genes that could be involved in directly determining the CR characteristics of ‘Hang Baishao’, and both of the genes need to be studied further.

### *HSP*s and *OSM*s increase bud resistance to natural cold and should receive more attention when mining the CR-associated genes

Some *HSP* genes (Unigene033982, Unigene001958, Unigene003118 and Unigene003256) were actively expressed from Dec. 10 to Dec. 24, and likely are involved in protecting dormant buds from cold stress. Numerous stress proteins such as HSPs (Table 3) can sensitively respond to temperature changes and enhance plant resistance as molecular chaperones under extreme heat shock stress. Chaperones protect inner proteins from denaturation and maintain the stability of these proteins [25, 54–56], and chaperones ultimately increase plant survivability to stress, including cold, heat, drought, strong salt-alkali or heavy metal ion stress [57–59].

Unigene033982 (*HSP18.2*) and Unigene010909 (*LOS1*) presented fluctuating patterns from November 2015 to February 2016; these patterns differed from those acquired from 2012 to 2013 (Fig. 4, Table 6). Homologs of *LOS1* are important for mRNA export under cold stress [60]. We did not find a distinct positive or negative correlation between the CR fulfillment of ‘Hang Baishao’ and the expression changes of *HSP18.2* or *LOS1* and could not confirm their linkage with CR characteristics as with *SOC1* and *WRKY33*. However, we should also pay enough attention to the genes coding for HSP or HSP-mediated proteins. These genes are often up-regulated during the endodormancy period because of low temperature during that time [7, 30, 61]. Also, the expression levels of these genes can be mediated or influenced by ABA and reactive oxygen species (ROS), thereby regulating the subsequent dormancy transition or prolonging the dormancy period, followed by a likely direct impact on the CR fulfillment period of ‘Hang Baishao’ [30, 55].

OSMs (Table 3) are members of the Pathogenesis-related Protein 5 (PR-5) family [62], and can strengthen plant membrane stability and resistance to multiple stresses [63–65]. The genes coding for OSMs or osmotin-like proteins (OLPs) impart increased resistance by up-regulating the expression of genes coding for ROS-scavenging enzymes and defense-related marker genes and participate in the cold acclimation of plants [66, 67]. In the present study, the expression of six *OSM* genes showed strong similarity. These patterns were not active during the coldest period but were clearly up-regulated on Jan. 21, 2013, after which they were down-regulated but then up-regulated again during the last period (Fig. 4). Unigene010777 (*ATOSM34*) showed high expression levels during the CR fulfillment period during both winters (Jan. 21, 2013 and Feb. 4, 2016) and could possibly be another closely CR-associated candidate gene.

### Genes that respond to photoperiod or regulate the circadian rhythm also need to be considered when mining the CR-associated genes

#### Photoperiod detection/signaling genes

In addition to temperature, photoperiod is another important natural cue for seasonal dormancy and sprouting [68], and is generally known as the primary signal for regulating endodormancy induction. In this research, *PAP2* (Unigene022263), *LHCB4.2* (Unigene017781) and other photoperiod/daylight-responsive genes sharply increased on Feb. 25, 2013 (Fig. 4). These genes and their homologs are sensitive to phytochrome signals [13, 68, 69] and regulate physiological activities induced by daylight or temperature during plant dormancy, development and flowering [8, 13, 27, 70]. With respect to ‘Hang Baishao’, high expression of these genes were correlated with the gradually extended day length during the late winter and early spring in Hangzhou. Only *PGK* (Unigene034695), *ATNADP*-*ME3* (Unigene006767) and *CAM8* (Unigene030455) genes had comparatively high expression levels from Dec. 24 to Jan. 21, which may be indirectly associated with the CR fulfillment of ‘Hang Baishao’.

### Circadian rhythm/clock genes

The circadian rhythm/clock is an important physiological phenomenon with respect to plant growth and flowering [71], and relevant circadian rhythm/clock genes are regulated by photoperiod and environmental temperature sensitivity [25, 72–74]. According to the RNA-Seq data, the genes that regulate the circadian rhythm, i.e., one *LHY*, one *TOC1* and two *APRR3* genes, were expressed highly from Dec. 10 to Jan. 21 of 2012–2013 (Fig. 4), which was the ecodormancy period with the shortest daylight and lowest temperature throughout the entire winter (Fig. 5). The active expression periods of Unigene005409 (*LHY*) and Unigene003485 (*TOC1*) were similar to those that occurred 3 years later, although the inner detailed expression patterns were different from Dec. 10 to Jan. 21 between the two winters (Fig. 4).

According to previous studies on the dormancy of *Populus* trees and leafy spurge, *LHY*, *TOC1* and *APRR3* may be conserved and could induce the expression of downstream genes in pathways that are closely related to dormancy release and flowering [12, 28, 75]. On the basis of its interaction, *TOC1* is probably a founding component of the gene circuit in the circadian clock of ‘Hang Baishao’ [74], and its expression in peony is consistent with its well described role in the positive regulation of *LHY* expression in other system; conversely, *LHY* may act as a negative element that represses *TOC1* expression [76]. Under low temperature, circadian oscillations are disrupted, which results in a high level of the constant expression of *LHY* or *TOC1*/*PRR1* [7]. Cold induction of *C-repeat binding factor* (*CBF*) may be positively regulated by the high expression of *LHY* or *TOC1*/*PRR1*, which can directly affect bud dormancy and freezing tolerance [7, 17, 77].

Interestingly, these four genes were sharply down-regulated after Jan. 21 during both winters. The day of Jan. 21 was a crucial time for ‘Hang Baishao’ for acquiring the sufficient CR in 2013 but not in 2016; therefore, temperature is not the most crucial impacting factor for these genes. Daylight duration and intensity are comparatively more stable during the months of winter each year compared with the instability of temperature during different winters. Down-regulation of these genes was likely influenced by day length extension after Jan. 21 when spring was approaching. Altered photoperiod and shortened or prolonged day length regulate the dormancy process in conjunction with temperature changes; therefore, the potential relationships and interactions among photoperiod-, temperature- and CR-associated genes are intriguing and deserve to be explored in future studies.

### A research system for mining candidate genes that regulate or determine the CR characteristics

**Fig. 7:**
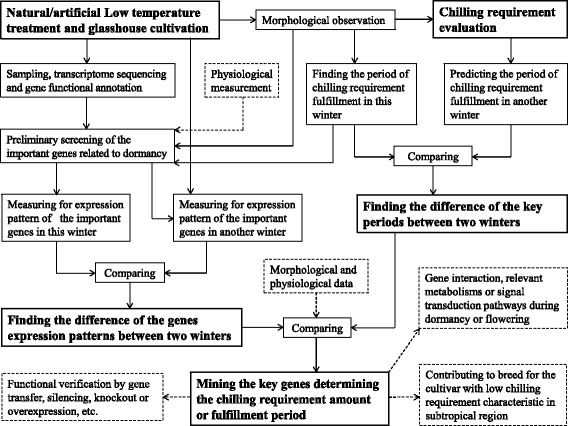
The research system for mining the candidate genes involved in regulating or determining the CR characteristic of herbaceous peony or other plants that undergo winter dormancy. The dotted boxes indicate the research work we have not carried out in this manuscript and will do in the future, or the practical significance of these researches

In the present study, we mainly focused on three aspects. First, we collected a large amount of morphological data for 2 years. These morphological observations were the important basis for subsequent in-depth research and deserved to be valued. Second, we selected two types of genes related to temperature and photoperiod as the main objects to excavate key genes that determine CR characteristics. We believe these two natural signals are primary and pivotal factors that regulate the CR amount and fulfillment period. In future studies, we will explore the key CR-associated genes from other types of genes, such as the genes related to water, carbohydrates, hormones and antioxidant metabolism (Fig. 6). Third, we focused on the consistencies between the differences of gene expression patterns and CR fulfillment periods in different winters. These consistencies could be important but not the conclusive evidence to suggest the decisive function of critical genes related to CR characteristics. Further verification using gene transfer, knockout, or overexpression will be performed in future research (Fig. 7). We developed a research system from this study and the data collection from several years in order to facilitate the understanding of the overall perspective for this study on peony, and provide a methodological reference for other researchers performing the similar studies.

## Conclusion

*P. lactiflora* ‘Hang Baishao’ has the characteristics of low chilling requirement and high adaptability to warm winters in the southern tropical region of China. Therefore, it should be a good material for CR research and pioneer parent for crossbreeding. The two genes of *SOC1* or *WRKY33* may be involved in determining the CR amount and fulfillment period of ‘Hang Baishao’, and *HSP*, *OSM*, *PAP2*, *LHY* and *TOC1* also deserve attention with respect to the exploration of CR-associated or CR-determining genes. After integrating all our research results from 2012 to 2016, we extracted a research system for mining genes that regulate or determine the CR characteristics of herbaceous peony or other perennials that undergo winter dormancy. These results and this system will provide useful information for further research on both key genes that regulate dormancy, cold response and CR characteristics. The results could also provide new insight into breeding new low-CR germplasm, and contribute to achieving the ultimate goal of “The southward plantation of herbaceous peony” in subtropical areas of the Northern Hemisphere.

## Additional files

Additional file 1: Table S1. Observations on sprouting and growth of ‘Hangbaishao’ after transferred into glasshouse under natural chilling treatments in 2012–2013. (XLSX 18 kb)

Additional file 2: Table S2. Observations on flowering performance of ‘Hangbaishao’ after transferred into glasshouse under natural chilling treatments in 2012–2013. (XLSX 270 kb)

Additional file 3: Table S3. The daily temperatures recorded in each hour from November 23, 2012 to March 12, 2013. (XLSX 73 kb)

Additional file 4: Table S4. Observations on sprouting and growth of ‘Hangbaishao’ after transferred into glasshouse under artificial chilling in 2013–2014. (XLSX 13 kb)

Additional file 5: Table S5. Observations on flowering performance of ‘Hangbaishao’ after transferred into glasshouse under artificial chilling treatments in 2013–2014. (XLSX 14 kb)

Additional file 6: Table S6. Details of the temperature- or phoroperiod-associated unigenes with differential expression acquired from transcriptomic sequencing. (XLSX 54 kb)

Additional file 7: Table S7. Details of the temperature- or phoroperiod-associated unigenes without differential expression from transcriptomic sequencing. (XLSX 443 kb)

Additional file 8: Table S8. The daily temperatures recorded in each hour from November 21, 2015 to March 15, 2016. (XLSX 149 kb)

## Abbreviations

ᅟ: The non-standard abbreviations mentioned in this manuscript are listed as follows. The Detail information about the abbreviations of morphological indices are elaborated in Supplementary 1, 2, 4 and 5. The gene abbreviations have been listed in Table 3which are not listed here again
ADF: Average Diameter of Flowers per flower
ADS: Average Diameter of mature and normal Stems per plant during the full flowering period
ALL: Average Leaf Length per plant during the full flowering period
ALT: Average Leaf Thickness per plant during the full flowering period
ALW: Average Leaf Width per plant during the full flowering period
ANA: Average Number of Aborted flowers per plant (NAF/3)
ANO: Average Number of Opening flowers per plant
ANS: Average Number of mature and normal Stems per plant during the full flowering period
AOD: Average Opening Days per flower
APH: Average Plant Height per plant during the full flowering period
APW: Average Plant Width per plant during the full flowering period
BBP: average Bud Break Percentage per replicate (seven plants/replicate) checked 5 weeks after being transferred into glasshouse
DEA: average number of Days per replicate between the transfer date and date of stem Elongated visibly for All plants
DFF: average number of Days per repliacate between the transfer date and date of the First plant Flowering
DFS: average number of Days per repliacate between the transfer date and date of the First plant Sprouting
FBP: average Final Bud break Percentage per replicate after being transferred into glasshouse.
NAB: average Number of Abnormal Buds per plant which sprouted and elongated, but finally developed into immature, abnormal or even withered stems (short, thin and delicate stems with few, small or abnormal leaves) during the full flowering period
NDF: Number of the sustaining Days of Full flowering (the number of days during PFF)
NOF: average Number of Opening Flowers per replicate
NPO: average Number of Plants per replicate with Opening flower
NSP: average Number of Small bud-Points per plant which could not sprout during the full flowering period in the year 2013
PAF: average Percentage of Aborted Flowers per replicate or per plant
PFF: Period of the Full Flowering (the period of over 50% flowers were opening simultaneously for three replicates of each sub-treatment)
POF: Average Percentage of Opening Flowers per replicate or per plant
PPO: Average Percentage of Plants with Opening flower per replicate (NPO/3)
RCC: average Relative Content of Chlorophyll of leaves
